# Antistaphylococcal and biofilm inhibitory activities of acetyl-11-keto-β-boswellic acid from *Boswellia serrata*

**DOI:** 10.1186/1471-2180-11-54

**Published:** 2011-03-16

**Authors:** Alsaba F Raja, Furqan Ali, Inshad A Khan, Abdul S Shawl, Daljit S Arora, Bhahwal A Shah, Subhash C Taneja

**Affiliations:** 1 Microbiology Unit, Indian Institute of Integrative Medicine (CSIR), Sanatnagar, Srinagar, 190005, India; 2 Clinical Microbiology Division, Indian Institute of Integrative Medicine (CSIR), Canal Road, Jammu, 180001, India; 3 Department of Microbiology, Guru Nanak Dev University, Amritsar Punjab143005, India; 4 Bioorganic Chemistry, Indian Institute of Integrative Medicine (CSIR), Canal Road, Jammu, 180001, India

## Abstract

**Background:**

Boswellic acids are pentacyclic triterpenes, which are produced in plants belonging to the genus *Boswellia*. Boswellic acids appear in the resin exudates of the plant and it makes up 25-35% of the resin. β-boswellic acid, 11-keto-β-boswellic acid and acetyl-11-keto-β-boswellic acid have been implicated in apoptosis of cancer cells, particularly that of brain tumors and cells affected by leukemia or colon cancer. These molecules are also associated with potent antimicrobial activities. The present study describes the antimicrobial activities of boswellic acid molecules against 112 pathogenic bacterial isolates including ATCC strains. Acetyl-11-keto-β-boswellic acid (AKBA), which exhibited the most potent antibacterial activity, was further evaluated in time kill studies, postantibiotic effect (PAE) and biofilm susceptibility assay. The mechanism of action of AKBA was investigated by propidium iodide uptake, leakage of 260 and 280 nm absorbing material assays.

**Results:**

AKBA was found to be the most active compound showing an MIC range of 2-8 μg/ml against the entire gram positive bacterial pathogens tested. It exhibited concentration dependent killing of *Staphylococcus aureus *ATCC 29213 up to 8 × MIC and also demonstrated postantibiotic effect (PAE) of 4.8 h at 2 × MIC. Furthermore, AKBA inhibited the formation of biofilms generated by *S. aureus *and *Staphylococcus epidermidis *and also reduced the preformed biofilms by these bacteria. Increased uptake of propidium iodide and leakage of 260 and 280 nm absorbing material by AKBA treated cells of *S aureus *indicating that the antibacterial mode of action of AKBA probably occurred via disruption of microbial membrane structure.

**Conclusions:**

This study supported the potential use of AKBA in treating *S. aureus *infections. AKBA can be further exploited to evolve potential lead compounds in the discovery of new anti-Gram-positive and anti-biofilm agents.

## Background

Nosocomial infections pose a significant threat to patients worldwide. Gram-positive bacterial pathogens are a significant cause of nosocomial infections that are important causes of morbidity and mortality [1]. Gram-positive bacterial pathogens such as *Staphylococcus aureus*, *Streptococcus pneumonia *and *Enterococcus faecalis *are clinically significant and the antibiotic resistance in these pathogens has become one of the major worldwide health problems. The emergence of methicillin-resistant *Staphylococcus aureus *(MRSA) and vancomycin-resistant *Enterococcus faecium *(VRE) are the major clinical concerns today [2]. The recent appearance vancomycin-intermediate resistant (VISA) and vancomycin-resistant *S. aureus *isolates (VRSA) in many countries is the latest development in antibiotic resistance [3]. MRSA has now exerted its own impact upon the mortality rate. The average mortality rate from a recent meta-analysis of 30 studies was ≈36% compared against a mortality rate of ≈24% from septicemia caused by methicillin-susceptible *S. aureus *[4].

Biofilms are communities of surface-associated microorganisms embedded in a self-produced extracellular polymeric matrix that are notoriously difficult to eradicate and are a source of many recalcitrant infections [5-9]. Staphylococci are known to form biofilms on an implanted medical device or damaged tissues and these biofilms are difficult to disrupt [10]. Biofilm infections are difficult to treat due to their inherent antibiotic resistance [11,12].

Boswellic acids are the major constituents of the gum derived from the plant *Boswellia serrata *Roxb. ex Colebr. (family Burseraceae, Syn. *B. glabra*). The gum resin comprises of β-boswellic acids as the main triterpenic acid along with 11-keto-β-boswellic acids and their acetates [13]. The gum exudate is known for its anti-inflammatory properties in the Ayurvedic system of medicines [14,15]. The alcoholic extract of the gum is used for the treatment of adjuvant arthritis [16]. It has synergistic effect with glucosamine, an anti-inflammatory and anti-arthritic agent [17]. Acetyl-11-keto-β-boswellic acid (AKBA), a component of the gum exudate is a pentacyclic terpenoid and is reported to be active against a large number of inflammatory diseases [18,19] including cancer, arthritis, chronic colitis, ulcerative colitis, Crohn's disease, and bronchial asthma [20-22]. In spite of these therapeutic effects of boswellic acids, little is known about their antibacterial activity and the active principle responsible. The aim of this study was to evaluate the antibacterial activity of acetyl-11-keto-β-boswellic acid and its effect on biofilms generated by *S. aureus *and *Staphylococcus epidermidis*.

## Results

### Minimum inhibitory concentrations (MIC) and minimum bactericidal concentrations (MBC) of boswellic acids

The *in vitro *antibacterial activities of boswellic acids were tested on a group of clinically significant Gram-positive and Gram-negative bacteria (Table 1). AKBA was the most active of the four boswellic acids against the bacterial pathogens. However the activity of AKBA was limited to Gram-positive bacteria only as its MIC was >128 μg/ml against *Escherichia coli *ATCC 25292 and *Pseudomonas aeruginosa *ATCC 27853 (Gram-negative pathogens used in this study). AKBA exhibited MIC ranging from 2-8 μg/ml against all the Gram-positive clinical isolates tested, whereas 11-keto-β-boswellic acid (KBA) and β-boswellic acid (BA) exhibited moderate Gram-positive antibacterial activity (MIC ≈ 8-64 μg/ml). Acetyl-β-boswellic acid (ABA) on the other hand was completely devoid of antibacterial activity upto the tested concentration of 128 μg/ml. All the compounds were bacteriostatic in nature and exhibited an MBC >128 μg/ml. Since AKBA was found to be the most active boswellic acid compound against Gram-positive bacterial pathogens, further *in vitro *studies were performed on this compound against clinically important *S. aureus *and *S. epidermidis*.

**Table 1:** Antibacterial activity of boswellic acid molecules against bacterial pathogens.

Organisms (CI^n^)		KBA	AKBA	BA	ABA	
		
	Ciprofloxacin	MIC^a^	MIC^a^	MIC^a^	MIC^a^	MBC^b^
*S. aureus *ATCC-29213	0.25	16	2	32	>128	>128
MRSA ATCC 3591, (50)	8->16	16-32	2-4	32-64	>128	>128
*E. faecalis *ATCC 29212, (22)	0.25-16	16-32	4-8	8-16	>128	>128
*E. faecium *ATCC 8042, (18)	0.25-16	16-32	4-8	8-16	>128	>128
*S. epidermidis *ATCC 12228,(12)	0.25->16	8-16	4-8	32-64	>128	>128
Vancomycin resistant *E. faecalis *(10)	>16	8-16	2-8	8-16	>128	>128
*E. coli *ATCC 25292	0.03	>128	>128	>128	>128	>128
*P. aeruginosa *ATCC 27853	0.12	>128	>128	>128	>128	>128

MICs and MBCs of boswellic acid molecules were determined using CLSI guidelines against 115 clinical isolates including ATCC strains. ^a^Minimum Inhibitory Concentration (μg/ml); ^b^Minimum Bactericidal Concentration (μg/ml); CI = Clinical isolates; n = number of clinical isolates.

### Postantibiotic Effect (PAEs)

The PAE of AKBA was determined on *S. aureus *ATCC 29213 (Table 2). The PAE induced by AKBA was concentration dependent, with duration 3.0 ± 0.1 h at 1 × MIC while at 2 × MIC it was 4.8 ± 0.1 h. Ciprofloxacin was used as control drug in the study and it exhibited a PAE of 1.4 ± 0.05 h at 1 × MIC while at 2 × MIC it was 2.2 ± 0.1 h (0.5 μg/ml). The PAEs of AKBA were significantly higher than the ciprofloxacin against *S. aureus *(*P *< 0.05).

**Table 2:** PAEs of Acetyl-11-keto-β-boswellic acid against *S. aureus *ATCC 29213.

Compounds	Mean PAE (h) ± SD on:	
	
	1 × MIC	2 × MIC
Acetyl-11-keto-β-boswellic acid	3.0 ± 0.1^a^	4.8 ± 0.1^b^
Ciprofloxacin	1.4 ± 0.05^a^	2.2 ± 0.1^b^

The PAEs were monitored by viable count of *S. aureus *after 2 h exposure to concentrations equal to MIC and 2 × MIC of antimicrobials (AKBA and ciprofloxacin). Values in the same column followed by the same superscripts are significantly different from each other (*P *< 0.05; Student's *t *test). PAE, Post antibiotic effect.

### Time-kill kinetic studies

**Figure 1:**
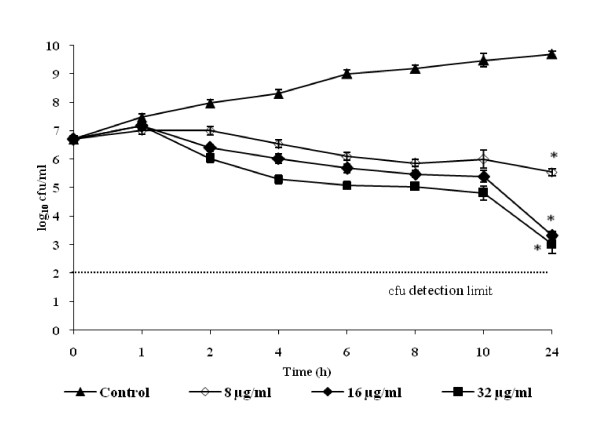
**Effect of AKBA at different concentrations (8, 16 and 32 μg/ml) on the cell viabilty of *S. aureus *ATCC 29213**. *S. aureus *cells without AKBA served as control. The effect of AKBA was observed bacteriostatic at all tested concentrations when compared with non treated control (*P *< 0.05) over a period of 24 h study. Each time point represents the mean log_10 _standard deviations (±SD) of three different experiments performed in duplicate. *, *P *< 0.05; (Student's *t *test).

The time-kill kinetic studies of AKBA were performed on *S. aureus *ATCC 29213 (Figure 1). It showed bacteriostatic activity at all the tested concentrations. The maximum effect of AKBA was observed at 16 and 32 μg/ml exhibiting a ≈2 log_10 _reduction in the viability of *S. aureus *cells when compared with non treated controls (*P *< 0.05) at four and eight times it's MIC over a period of 24 h study.

### Biofilm inhibition and reduction

**Figure 2:**
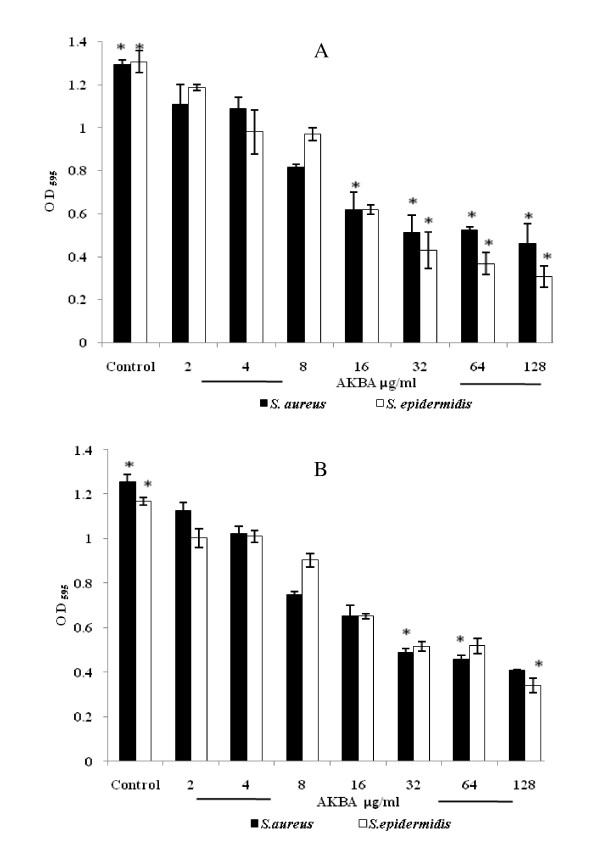
**Effect of AKBA on the biofilm formation (A) and preformed biofilm (B) by *S. aureus *ATCC 29213 and *S. epidermidis *ATCC 12228**. After incubation, the biofilms were stained with crystal violet and the optical density of stained adherent bacteria was determined with a multidetection microplate reader at a wavelength of 595 nm (OD_595_). The results are expressed as average optical density readings for crystal violet assays compared to growth control. The biofilm of *S. aureus *and *S. epidermidis *were significantly inhibited (A) and reduced (B) compared with those of bacteria without AKBA (*P *< 0.01). Values are mean (±SD) from four independent determinations. *, *P *< 0.01 (Student's *t *test).

AKBA effectively inhibited the formation of *S. aureus *and *S. epidermidis *biofilms, with 50% biofilm inhibition concentration (MBIC_50_) from 16-32 μg/ml (as derived from Figure 2A) which is in the range of 4 × MIC and 8 × MIC respectively. AKBA also effectively eradicated the preformed biofilms. The 50% biofilm reduction concentration (MBRC_50_) ranged from 32-64 μg/ml for both the bacterial isolates (Figure 2B).

### Effect of AKBA on membrane integrity

**Figure 3:**
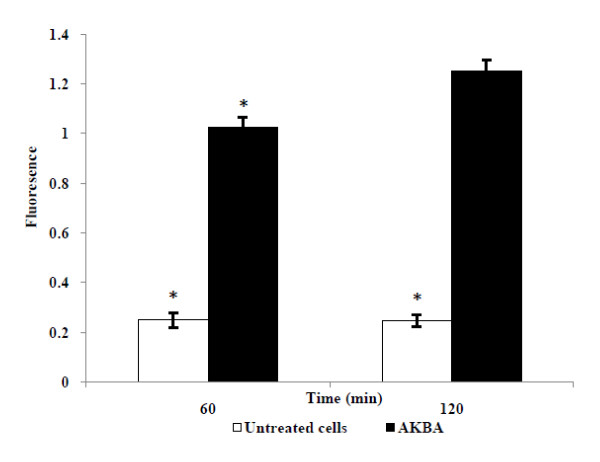
**Uptake of propidium iodide in cell of *S. aureus *ATCC 29213**. Cells of *S. aureus *were treated with AKBA at 64 μg/ml for 60 and 120 min. Control group included cells untreated with AKBA. AKBA treated cells significantly increases the fluorescence compared with untreated control (*P *< 0.05). Data represent the mean and standard deviations (±SD) of two different experiments performed in triplicate. *, *P *< 0.05 (Student's *t *test).

**Figure 4:**
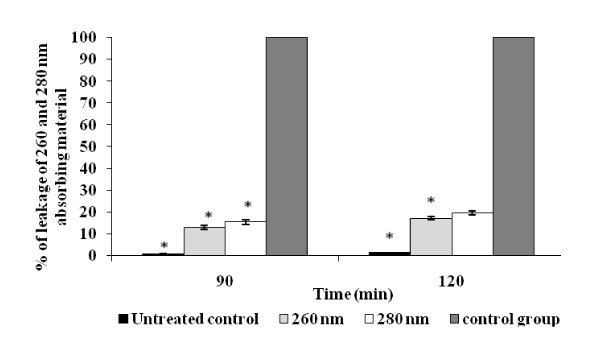
**Effect of AKBA on the leakage of 260 and 280 nm absorbing materials in *S. aureus *ATCC cells**. Control group (treated with lytic enzymes and considered as 100% leakage) and treated with AKBA at 64 μg/ml for 90 and 120 min. No compound added served as untreated control. Values are means (±SD) from three independent determinations. *, *P *< 0.05 (Student's *t *test), AKBA treated group compared to untreated control group.

In order to investigate the antibacterial action of AKBA on the bacterial membrane integrity, the cell suspension of *S. aureus *ATCC 29213 was exposed to a concentration of 64 μg/ml AKBA for 60 and 120 min followed by staining with propidium iodide (nucleic acid stain). The AKBA exposure resulted in bacterial cell membrane disruption as evident from the increased uptake of propidium iodide in comparison to the unexposed cells (*P *< 0.05) (Figure 3). In addition, the membrane leakage assay illustrated the cytoplasmic membrane damage of *S. aureus*. The amount of 260 and 280 nm absorbing material in *S. aureus *cell supernatants treated with AKBA (relative to the total released upon complete cell lysis) was 12 and 15% at 90 min while it was 15 and 19% at 120 min respectively (Figure 4), which was significantly higher than the untreated control (*P *< 0.05).

## Discussion and conclusion

The gum exudate or the resin obtained from the bark of *Boswellia serrata *has been widely used by the practitioners of the Indian systems of medicine for various medical conditions such as arthritis, asthma, ulcers, and skin diseases; currently it is being extensively used in various formulations for the treatment of inflammation related disorders [13-15]. The major chemical components of gum resin can be divided into three groups: volatile oils or lower terpenoids, higher terpenoids, and carbohydrates. The higher terpenoids comprises of β-boswellic acids as the main triterpenic acid along with 11-keto-β-boswellic acids and their acetates [23].

The *in vitro *antibacterial activity results of four boswellic acid compounds revealed AKBA to be the most potent antibacterial compound against Gram-positive pathogens, but it showed no significant antibacterial activity (MIC >128 μg/ml) against the Gram negative bacteria. AKBA exerted bacteriostatic antibacterial activity against S. *aureus *ATCC 29213 (Figure 1) and exhibited a good PAE of 4.8 h at 2 × MIC concentration. Staphylococci cause a large percentage of catheter associated infections, and like many other pathogens, rather than living as free planktonic cells within the host they tend to form a multilayered community of sessile bacterial cells known as a biofilm on medical implants or damaged tissue [7,24,12]. Biofilm infections are difficult to treat due to their inherent antibiotic resistance [7,12,25]. AKBA effectively inhibited the staphylococcal biofilm and also reduced the preformed biofilm of these bacterial pathogens (*P *< 0.01). To our knowledge, this is the first report to provide the evidence that AKBA can prevent as well as reduce the *S. aureus *and *S. epidermidis *generated biofilms.

AKBA is reported to be active against a large number of inflammatory diseases, cancer, arthritis, chronic colitis, ulcerative colitis, Crohn's disease, and bronchial asthma [21,26,20,28]. The anticancer activity of AKBA is attributed to the inhibitory effect on the lipoxygenases leading to the inhibition of cell proliferation and induction of apoptosis in tumor cells [29]. There are numerous reports available on the antibacterial activity of oleo-gum resin extracts and oleo-gum resin essential oils from *Boswellia *spp. (Burseraceae) [30-32]. Weckessera et al. [33] reported the antibacterial activity of *Boswellia *dry extract and keto-ß-boswellic acid. Their findings revealed that the extract was highly effective against selected aerobic and anaerobic bacteria such as *Streptococcus*, *Corynebacteria*, *C. perfringens *and *P. acnes*; whereas KBA was not effective against these pathogens, suggesting that the effective components are other boswellic acids or essential oils contained in the extract. In this study, we extensively evaluated the boswellic acids for the antibacterial activity and further for the first time established that AKBA is the single most potent antibacterial compound present in the gum exudates of *Boswellia serrata*.

We further investigated the effect of AKBA on the bacterial cell membrane integrity through propidium iodide uptake assay. Propidium iodide is fluorescent nucleic acid stain that binds to DNA by intercalating between the bases with little or no sequence preference. It is membrane impermeant and generally excluded from viable cells. The increased uptake of propidium iodide in the AKBA treated cells of *S aureus *in our study indicated that AKBA altered the cell membrane structure, resulting in the disruption of the permeability barrier of microbial membrane structures. Leakage of cytosolic constituents (260 and 280 nm absorbing materials) from *S. aureus *cells in the presence 64 μg/ml AKBA over a period of two h was significantly higher than background levels (*P *< 0.05). These observations indicate that the antimicrobial activity of AKBA results from its ability to disrupt the permeability barrier of microbial membrane structures. The lack of antibacterial activity of AKBA against Gram-negative bacteria may be attributed due to the presence of lipophilic outer membrane. This outer layer of the Gram-negative outer membrane is composed primarily of lipopolysaccharide molecules and forms a hydrophilic permeability barrier providing protection against the effects of highly hydrophobic compounds [34,35]. This may be the probable explanation of the resistance of Gram-negative bacteria to lipophilic AKBA. Similar observations have been made in other studies also, where lipohilic terpenes such as carvacrol, thymol, eugenol, geraniol, linalyl acetate, (-) menthol and bakuchiol have reported low sensitivities against Gram-negative bacteria [36-38].

Gum resin of *boswellia *is included in the list of substances Generally Recognized As Safe (GRAS), thereby permitting its use as food additive by US FDA. Boswellic acid extract and AKBA have also been reported to be safe and exert minimal toxicity on human skin cells [39]. The recent study indicates that *B. serrata *is non-mutagenic in Ames test, and is non-clastogenic in *in vitro *chromosomal aberration study [40]. Oral preparations of *Boswellic serrata *extract containing AKBA are sold in the market as over the counter (OTC) anti-inflammatory formulations and are considered to be quite safe [41]. The ancient Indian system of medicine (Ayurveda) claims these preparations to be safe and effective dietary supplement against joint disorders [42,14,15]. Preliminary pharmacokinetic studies carried out in humans yielded low concentrations of boswellic acids in plasma [43-45]. In the study reported by Buechele and Simmet [44] AKBA was found in plasma at a concentration of 0.1 μM after the daily intake of 4 × 786 mg *Boswellia *extract for 10 days. In accordance with the observations made in humans, KBA and AKBA were detected at a concentration of 0.4 and 0.2 μM, respectively; in rat plasma following single oral dose administration of 240 mg/kg *Boswellia serrata *extract [46]. Further attempts should be made to improve the bioavailability of AKBA through lipid based delivery systems. As the literature suggested that the intake of a high fat meal increases three to fivefold in the plasma concentrations of boswellic acid molecules [47].

In addition to the above reported usage and safety associated with AKBA, the potent antibacterial activity reported in this study warrants that the structure of AKBA can be further exploited to evolve potential lead compounds in the discovery of new anti-Gram-positive and anti-biofilm agents.

## Methods

### Extraction and isolation of boswellic acid molecules from gum resin of *Boswellia serrata*

BA, KBA, ABA and AKBA were obtained from Bio-organic Chemistry Division of Indian Institute of Integrative Medicine Jammu, India. The extraction, isolation, and quantification of these compounds from gum resin of *Boswellia serrata *were described in our previous study [17,23].

### Bacterial strains and culture conditions

The bacterial strains used in this study were *S. aureus *ATCC 29213, methicillin-resistant *S. aureus *(MRSA) ATCC 33591, *E. faecalis *ATCC 29212, *E. faecium *ATCC 8042, S. *epidermidis *ATCC 12228, *E. coli *ATCC 25292, *P. aeruginosa *ATCC 27853 and 112 isolates of various bacterial pathogens (MRSA 50, *E. faecalis *22, *E. faecium *18, *S. epidermidis *12 and vancomycin resistant *E. faecalis *10). All ATCC strains were procured from the American Type Culture Collection (ATCC, Manassas, VA, USA). Clinical isolates of all strains were kindly gifted by Ranbaxy Laboratories Limited, India and Lupin pharmaceutical, Pune, India. The cultures were stored at -70°C in trytone soya broth containing 50% glycerol (vol/vol; Himedia, Mumbai India) and maintained on tryptic soy agar (TSA; Difco Laboratories, Detroit, Mich USA).

### Determination of minimum inhibitory concentration (MIC) and minimum bactericidal concentrations (MBC)

MIC was determined as per the guidelines of Clinical and Laboratory Standards Institute (formerly the National Committee for Clinical Laboratory Standards) [48]. Briefly, the bacterial suspensions were prepared by suspending 18 h grown bacterial culture in sterile normal saline (0.89% NaCl wt/vol; Himedia, Mumbai India). The turbidity of the bacterial suspension was adjusted to 0.5 McFarland standards (equivalent to 1.5 × 10^8 ^colony forming units (CFU)/ml). The boswellic acids stock solutions were prepared in 100% dimethyl sulfoxide (DMSO; Merck, Mumbai India) and 2-fold serial dilutions were prepared in Mueller Hinton Broth (MHB; Difco Laboratories) in 100 μl volume in 96-well U bottom microtiter plates (Tarson, Mumbai, India). The above-mentioned bacterial suspension was further diluted in the MHB and 100 μl volume of this diluted inoculum was added to each well of the plate resulting in the final inoculum of 5 × 10^5 ^CFU/ml in the well and the final concentration of boswellic acids ranged from 0.25 to 128 μg/ml. Ciprofloxacin was used as standard antibacterial agent for this study at a concentration ranged from 0.03-16 μg/ml. The plates were incubated at 37°C for 18 h and were visually read for the absence or presence of turbidity. The minimum concentration of the compound concentration showing no turbidity was recorded as MIC. The MBC was determined by spreading 100 μl volume on tryptic soy agar (TSA) plate from the wells showing no visible growth. The plates were incubated at 37°C for overnight.

### Time kill assay

*S. aureus *ATCC 29213 was grown in MHB at 37°C for 24 h. The turbidity of the suspension was adjusted to 0.5 McFarland standard (≈ 1.5 × 10^8 ^CFU/ml) in sterile normal saline. Two hundred microliters of this suspension was used to inoculate 20 ml of MHB in conical flasks containing AKBA in the concentration range of 8-32 μg/ml. DMSO controls were also included in the study. The flasks were incubated at 37°C. One hundred microliters samples were taken at 0, 1, 2, 4, 6, 8, 10, and 24 h and the viable counts were determined in triplicate on TSA. Killing curves were constructed by plotting the log_10 _CFU/ml versus time over 24 h [49].

### Postantibiotic Effect (PAE)

The PAEs of the AKBA were assessed by the method described by Craig and Gudmundsson [50]. AKBA was added at the MIC and 2 × MIC to test tubes containing ≈10^6 ^CFU/ml of *S. aureus *ATCC 29213 in MHB broth. After an exposure of 2 h to the AKBA, samples were diluted to 1:1,000 in same medium to effectively remove AKBA. CFU was determined from the sample every hour until visual cloudiness was noted. The PAE was calculated by the equation: PAE = *T - C*, where *T *represents the time required for the count in the test culture to increase 1 log_10 _CFU/ml above the count observed immediately after drug removal and *C *represents the time required for the count of the untreated control tube to increase by 1 log_10 _CFU/ml.

### Biofilm susceptibility assay

The biofilms of *S. aureus *ATCC 29213 and *S. epidermidis *ATCC 12228 were prepared in 96-well flat-bottom polystyrene microtiter plates (Tarson, Mumbai, India), using a previously described method of Wei et al. [51] with a few modifications. This method was similar to the MIC assay for planktonic cells. The bacterial suspensions were prepared from the overnight grown culture and the turbidity of the suspension was adjusted to 0.7 O.D._610 _(≈1 × 10^9 ^CFU/ml). Twofold serial dilutions of boswellic acids were prepared in 100 μl volume in tryptone soya broth (TSB; Difco laboratories) supplemented with 0.5% glucose in the wells of 96-well flat bottom microtiter plate. Forty microliters of fresh TSB with 0.5% glucose was added to each well, followed by the addition of 60 μl of above bacterial suspension. This resulted in the final inoculum of 6 × 10^7 ^CFU/ml in each well: the final concentrations of the compounds ranged from (0.12 to 128 μg/ml). The plate was incubated for 18 h at 37°C. After completion of incubation, the planktonic cells were removed from each well by washing with phosphate buffer saline (Himedia, Mumbai, India). The biofilms were fixed with methanol for 15-30 min, stained with 0.1% (wt/vol) Crystal Violet (Sigma Chemical Co., St Louis, MO, USA) for 10 min and rinsed thoroughly with water until the negative control wells appeared colorless. Biofilm formation was quantified by the addition of 200 μl of 95% ethanol to the crystal violet stained wells and recording the absorbance at 595 nm (A_595_) using a microplate reader (Multiskan spectrum, Thermo electron, Vantaa, Finland).

The effect of AKBA was also examined on preformed biofilms. The biofilms were prepared by inoculating the suspension of *S. aureus *and *S. epidermidis *into the wells of a polystyrene microtiter plate as mentioned above. After incubation at 37°C for 18 h, the culture supernatant from each well was decanted and planktonic cells were removed by washing the wells with PBS (pH 7.2). Two fold serial dilution of AKBA was prepared in TSB and 200 μl of each dilution was added to the biofilm in the wells. The plate was further incubated at 37°C for 18 h. The biofilm was fixed, stained and quantified as described above.

### Propidium iodide uptake assay

The action of AKBA on cell membrane permeability of *S. aureus *ATCC 29213 cells was evaluated by the method as described by Cox et al. [52]. The bacterial cells were grown overnight in 100 ml of MHB at 37°C, washed and resuspended in 50-mmol/l sodium phosphate buffer, pH 7·1. The turbidity of the suspension was adjusted to 0.7 O.D._610 _(≈1 × 10^9 ^CFU/ml). One milliliter volume of this suspension was added to flask containing 19 ml buffer and 64 μg/ml of AKBA. Following 60 and 120 min incubation at room temperature, 50 μl aliquots were transferred into Eppendorfs tubes containing 950 μl phosphate buffer in FACS tubes (Becton Dickinson Biosciences, CA, USA). These tubes were stored on ice and 5 μl of staining solution, consisting of 2.5 mg/ml propidium iodide (Sigma) dissolved in milliQ water, was added in the final propidium iodide concentration of 10 μg/ml. The cells were subjected to FACS analysis [53,54], on the flow cytometer (BD-LSR, Becton Dickinson).

### Leakage of 260 and 280 nm absorbing compounds

The release of 260 and 280 nm absorbing compounds was determined spectrophotometrically [55]. Briefly, cells suspensions of *S. aureus *were prepared as for propidium iodide uptake assay. AKBA was added at 64 μg/ml to the bacterial suspension (≈1 × 10^9 ^CFU/ml) and incubated for 120 min at 37°C. For the complete release of 260 and 280 nm absorbing compounds, the bacterial suspension (control) was treated with lysozyme (100 μg/ml) at 37°C for 120 min, followed by sonication. Cell supernatants were obtained by centrifugation (10,000 g for 10 min). The absorbance of cell supernatant at 260 and 280 nm was determined using spectrophotometer (Multiskan Spectrum). Background leakage rates (no compounds added) were used as untreated control. The extent of leakage of 260 and 280 nm absorbing compounds was expressed as percentage of control (suspension treated with lysozyme) measured in supernatants.

### Statistical analysis

All experiments were carried out in triplicates in at least three different occasions. Differences between two means were evaluated by the Student's *t*-test. The data were analyzed by one-way ANOVA for comparison of multiple means followed by post bonferroni test using GraphPad Instat2 program (GraphPad software Inc. San Diego CA). The chosen level of significance for all statistical tests was *P  *< 0.05.

## Authors' contributions

AFR, FA and IAK have made substantial contributions to conception and design, acquisition of data, analysis and interpretation of data. ASS and DSA have been involved in drafting the manuscript and revising it critically for important intellectual content. BAS and SCT provided the all four Boswellic acid molecules. All Authors helped to draft the manuscript, participated sufficiently in the work to take public responsibility for appropriate portions of the content and approved the final manuscript.
